# Association of caesarean scar defect with risk of abnormal uterine bleeding: results from meta-analysis

**DOI:** 10.1186/s12905-024-03198-6

**Published:** 2024-07-29

**Authors:** Xiao-Jing Xu, Jing-Xing Jia, Zi-Qiong Sang, Li Li

**Affiliations:** 1 Hubei Minzu University, Enshi, Hubei 445000 China; 2 Department of Obstetrics, Minda Hospital of Hubei Minzu University, No.2 of Wufengshan Road, Tuqiao Road, Enshi, Hubei 445000 China

**Keywords:** Caesarean scar defects, Abnormal uterine bleeding, Meta-analysis

## Abstract

**Objective:**

To investigate the association between caesarean scar defects and abnormal uterine bleeding through systematic literature review.

**Methods:**

PubMed, Web of Science, Cochrane Library and Embase databases were searched based on PRISMA 2020 to include studies exploring abnormal uterine bleeding in women with caesarean scar defects. The combined relative risk (RR) of uterine bleeding, combined prevalence of abnormal uterine bleeding and combined RR of intermenstrual uterine bleeding were calculated using a fixed- or random-effects model.

**Results:**

Ten studies involving 1,183 women with caesarean scar defects met the inclusion criteria for this study. Compared with women without caesarean scar defects, those with caesarean scar defects had a higher risk of abnormal uterine bleeding (RR: 3.22, 95% CI: 1.83–5.66) and intermenstrual bleeding (RR: 2.93, 95% CI: 1.91–4.50). The prevalence of abnormal uterine bleeding was approximately 0.46 (95% CI: 0.27–0.64), and across populations, women with a previous caesarean section who had undergone imaging specifically for gynaecological disease had a significantly higher prevalence of abnormal uterine bleeding (0.77, 95% CI: 0.65–0.89) than those with at least one caesarean Sect. (0.25, 95% CI: 0.10–0.39).

**Conclusion:**

A significant association was observed between caesarean scar defects and abnormal uterine bleeding, with the former being a risk factor for the latter. However, previous studies have differed in the definition of caesarean scar defects and abnormal uterine bleeding, and more high-quality studies are needed to further investigate the relevant definitions and study results in the future.

## Introduction

Caesarean section is an effective and important obstetric surgery that can save the lives of both mothers and infants. Epidemiological data show that the caesarean section rate is gradually rising worldwide, approximately doubling since 2000, with a global rate of around 21.7%; this is despite the World Health Organisation’s recommendation that the caesarean section rate should remain around 10% [1–3]. In China, the caesarean section rate was as high as 32.7% between 2008 and 2014 [4]. The increasing rate has also led to growing concerns about the long-term complications of caesarean section. After lower segment caesarean section, some patients’ scars at the uterine incision site develop into concave anatomical defects, known as caesarean scar defects or caesarean scar diverticula [5]. Previous studies have shown that caesarean scar defects occur in up to 70% of women undergoing caesarean section, 30% of whom experience symptoms [6].

Over the past few decades, numerous studies have been conducted on caesarean scar defects, which may lead to gynaecological symptoms such as abnormal uterine bleeding [7], pain [8], secondary infertility [9] and asymptomatic uterine rupture [10]. The possible mechanisms of abnormal uterine bleeding associated with caesarean scar defects may include menstrual blood retention in the defect, impaired drainage of fibrous tissue and blood production within the defect due to the presence of neovascularisation, inflammation and adenomyosis [11, 12]. The standard treatment has not yet been established for the management of caesarean scar pregnancy; therefore, establishing an understanding of abnormal uterine bleeding and caesarean scar defects is important to facilitate clinical practice [13]. However, there is a lack of systematic reviews on the risk of abnormal uterine bleeding in women with caesarean scar defects. This study is designed to systematically review the existing literature to determine the relationship between caesarean scar defects and the risk of abnormal uterine bleeding, thus providing an evidence-based reference for clinical practice in this field.

## Materials and methods

### Retrieval strategy

Following the PRISMA 2020 statement [14], literature was systematically retrieved from four English databases, namely, PubMed, Web of Science, Cochrane Library and Embase, from their inception to November 30, 2023. The retrieval strategies included the following keywords: ‘Cesarean scar’, ‘caesarean scar’, defect’, ‘isthmocele’, ‘Cesarean scar dehiscence’, ‘uterine diverticulum’, ‘niche’, ‘Cesarean scar pouch’, ‘abnormal bleeding’, ‘abnormal uterine bleeding’ and ‘bleeding’. Additionally, targeted articles were obtained by reviewing relevant reviews and references from included studies.

### Inclusion and exclusion criteria

The inclusion criteria were as follows: (1) studies published in peer-reviewed journals in English or Chinese; (2) studies involving subjects with caesarean scar defects (defined by the investigators); (3) outcomes of interest, including the risk or prevalence of uterine bleeding, menstrual bleeding or abnormal uterine bleeding; different definitions of abnormal uterine bleeding were allowed, but mainly focused on intermenstrual bleeding, postmenstrual bleeding and unscheduled bleeding; and (4) cross-sectional studies, cohort studies and case-control study designs.

The exclusion criteria included: (1) non-population-based studies; (2) conference articles, case reports, systematic reviews and other non-original studies; (3) insufficient outcome data for data analysis; (4) duplicate publications of the same study; and (5) studies for which full-text articles could not be obtained.

### Literature screening and data extraction

Literature screening was performed as per the inclusion and exclusion criteria by two investigators separately, first by reading the titles and abstracts of the articles for initial screening, followed by a full-text reading of articles that met the inclusion criteria. In cases of disagreement between the two investigators, a third investigator was consulted, with a consensus reached upon discussion. Following literature screening, the two investigators independently extracted data using a standardised data extraction form, including publication information, demographic characteristics of the study participants, study time and outcome events.

### Quality evaluation

The Newcastle–Ottawa Scale (NOS) [15] was used to evaluate the quality of cohort studies and case-control studies. Eight items were evaluated, including representativeness of the study population, comparability between groups, adequacy of outcome evaluation, sufficiency of follow-up time and completeness of follow-up. The maximum score was 9, with a score of 7 or above indicating high-quality articles and a score of 5 or below indicating low-quality articles. Moreover, the quality of cross-sectional studies was evaluated using the JBI Quality Appraisal Checklist [16], which consists of nine evaluation items, and each item is scored as 1 if the included article meets the criteria. The maximum score is 9.

### Statistical analysis methods

Statistical analysis was performed using Stata 16.0 software. The effect size for count data was expressed as relative risk (RR), with the 95% confidence interval (CI) used for estimating the interval range. Heterogeneity was determined using *I*^*2*^ statistics and the Q test: *I*^*2*^ < 50% or *P* > 0.1 indicated homogeneity among the included articles, and a fixed-effects model was used for analysis; *I*^*2*^ > 50% or *P* < 0.1 indicated poor homogeneity among the included articles, in which case a random-effects model was used for analysis. For significant heterogeneity, subgroup analyses or sensitivity analyses were conducted to explore the source of heterogeneity. Unless otherwise specified, the significance level was set at 0.05.

## Results

### Basic characteristics and quality evaluation of included articles

**Fig. 1:**
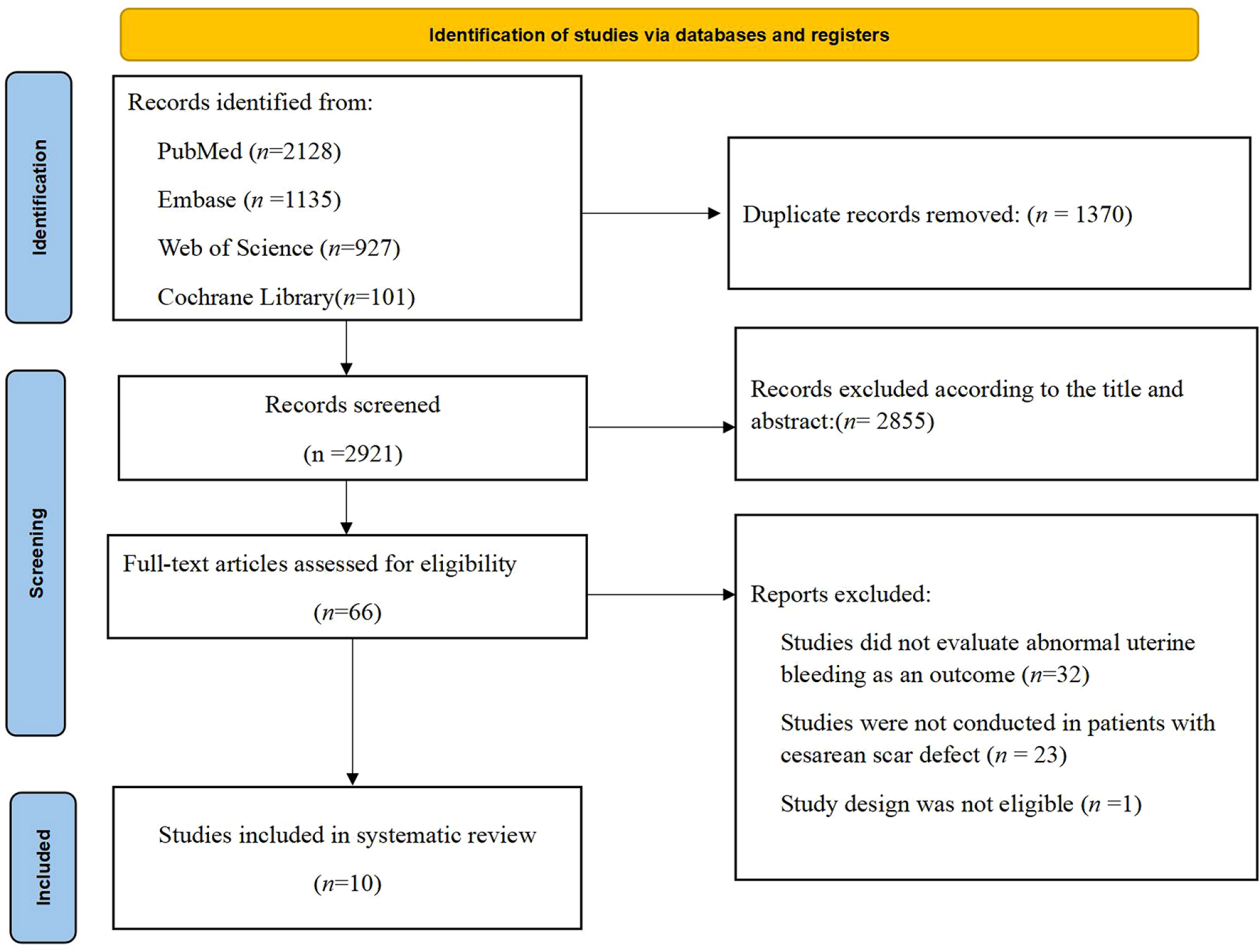
Flow chart of literature screening

After excluding 1,370 duplicate articles, a preliminary screening of the titles and abstracts of 2,921 articles was conducted, 66 articles were included for full-text review, and finally, 10 articles [11, 17–25] met the inclusion criteria for this study. The literature screening workflow is shown in Fig. 1. The eligible articles were published between 2001 and 2021 from countries including the United States, China, Finland, Italy, the Netherlands and Chile. The study designs included cohort studies, case-control studies and cross-sectional studies, involving 1,183 subjects with caesarean scar defects, and there was some variability in the definitions of caesarean scar defects and abnormal uterine bleeding among these studies. The quality evaluation results suggested a high quality of the included articles. See Table 1 for more data on the basic characteristics and quality evaluation results of the included articles.

**Table 1:** Characteristics of included studies

Author, Year	Study Design	Location	Population	Definition of CSD	Definition of AUB	Patients with CSD	Age	NOS/JBI score
Monteagudo,2001	cohort study	USA	women with a history of CS presenting for imaging with various gynecologic indications	Triangular anechoic filling defect in the anterior wall of the uterus	unscheduled or heavy menstrual bleeding	44	< 30: 6 31 − 40: 18 41-50: 17 > 50: 3	7
Fabres,2003	cohort study	Chile	premenopausal women with histories of at least 1 cesarean delivery	filling defect of the uterine cavity located in relation to the anterior isthmus	postmenstrual spotting and midcycle metrorrhagia	92	39/38	7
Menada,2006	case-control	Italy	women with a history of CS or vaginal birth	triangular, anechoic structure at the presumed site of CS	spotting bleeding after the end of the menstrual on and/or non-cyclic bleeding not related to the menstrual on	116	30.9/31.4	8
Wang,2009	cross-sectional	China, Taiwan	women with a history of cesarean section screened using transvaginal ultrasound for various gynecological indications	presence of a hypoechogenic area (a filling defect) within the myometrium of the lower uterine segment	postmenstrual spotting	207	35.2	6
Bij,2011	cohort study	Netherlands	consecutive women who had a cesarean section	an anechoic area at the site of the cesarean scar with a depth of at least 1 mm	intermenstrual bleeding, and postmenstrual spotting	117	35.2/34.5	9
Li,2014	cross-sectional	China	women who had previous cesarean deliveries	triangular anechoic structure at the presumed site of the incision	postmenstrual abnormal uterine bleeding	41	34.8	8
Van der Voet,2014	cohort study	Netherlands	women aged over 18 years of age who underwent a cesarean section	anechoic space (with or without fluid) at least 2 mm deep at the presumed site of the cesarean section scar	postmenstrual spotting	129	32.66/32.26	8
Van der Voet,2017	cohort study	Netherlands	women with a request for hysteroscopic sterilization	any visible defect, disruption, or concavity (gap) in the anterior wall	intermenstrual bleeding	83	38.4/39	8
Antila,2020	cohort study	Finland	women who delivered by cesarean section	anechoic defect at least 2.0 mm deep at the site of the CS scar	postmenstrual spotting which was defined as at least 2 days of brownish discharge after the end of the menstrual period	145	33.4/32.0	9
Zhou,2021	cohort study	China	women with a history of at least one cesarean section	presence of fluid within the scar	postmenstrual spotting, prolonged menstruation, and continuous brown discharge	209	34.44/35.04	8

*Note* CS, cesarean delivery; CSD, cesarean scar defect; AUB, abnormal uterine bleeding; NOS, Newcastle-Ottawa Scale

### Risk of abnormal uterine bleeding

**Fig. 2:**
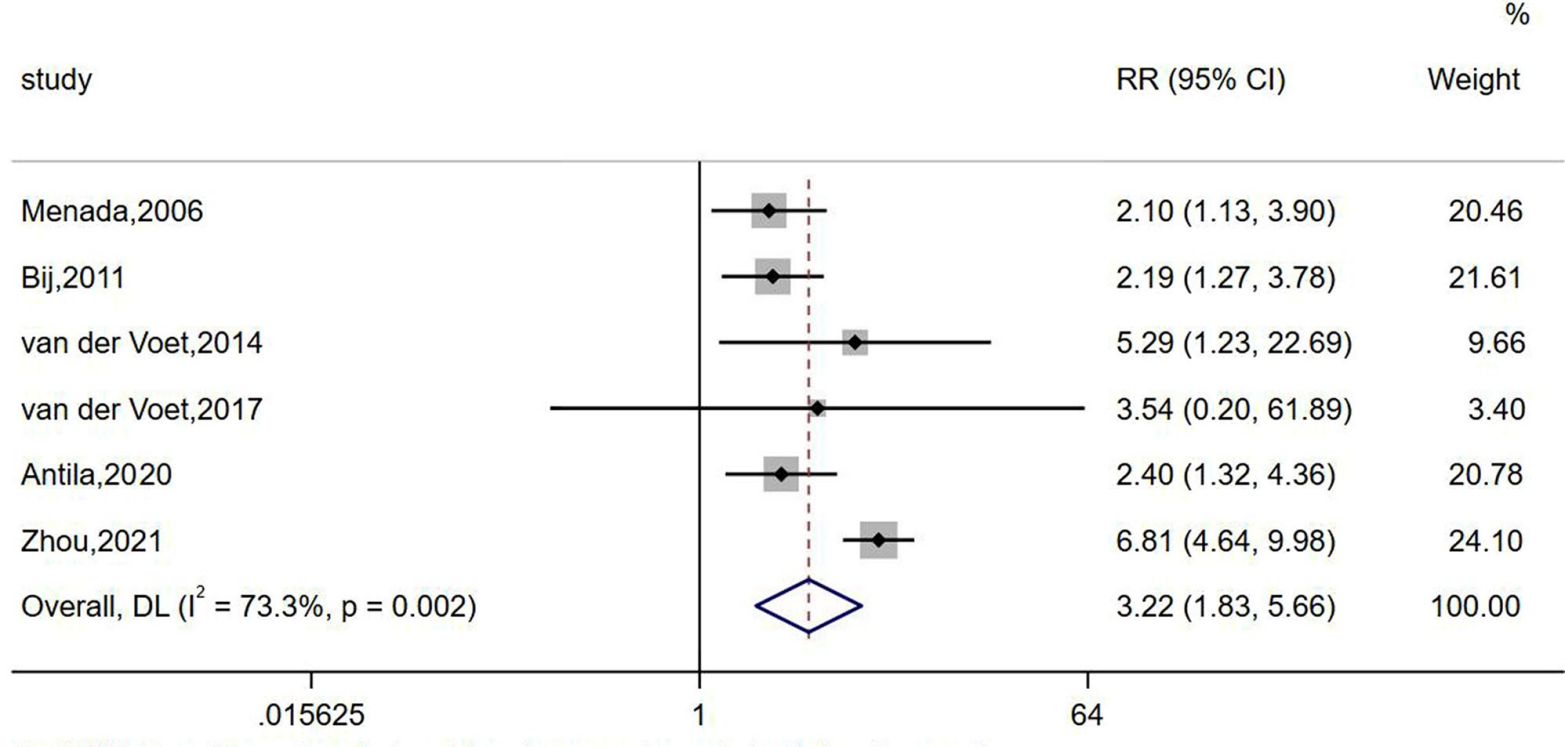
Forest plot of the relative risk of abnormal uterine bleeding in patients with Cesarean scar defect versus those without

**Fig. 3:**
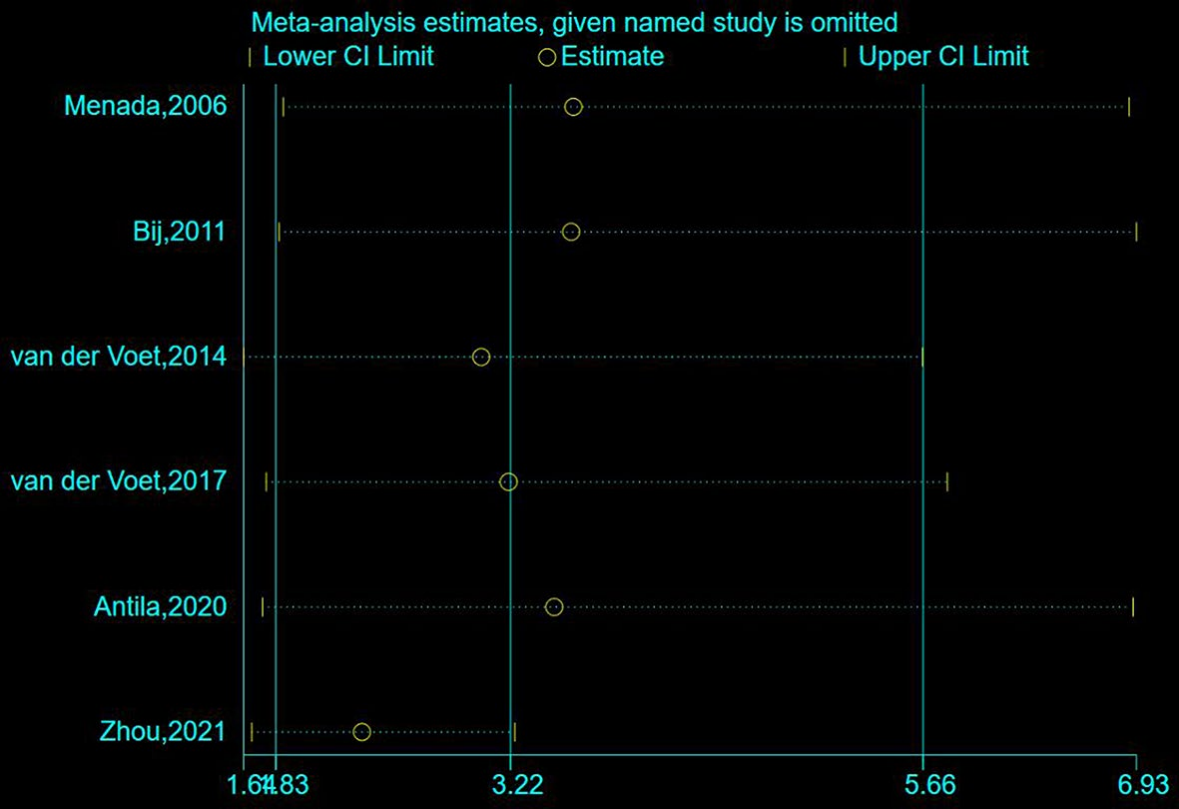
Sensitivity analysis of the relative risk of abnormal uterine bleeding in patients with Cesarean scar defect versus those without

Six studies compared the risk of abnormal uterine bleeding in women with/ without caesarean scar defects, involving a sample size of 761 exposed participants and 798 controls. Pooled risks were calculated using a random-effects model based on the heterogeneity evaluation (*I*^*2*^ = 73.3%, *P* = 0.002). The meta-analysis showed that subjects with caesarean scar defects had a 3.22-fold increased risk of abnormal uterine bleeding (95% CI: 1.83–5.66) compared with the control group, as shown in Fig. 2. Since heterogeneity was observed among the included articles, sensitivity analyses were conducted to identify potential sources of heterogeneity; however, no clear sources were found after excluding the included articles one by one, as shown in Fig. 3.

### Prevalence of abnormal uterine bleeding

**Fig. 4:**
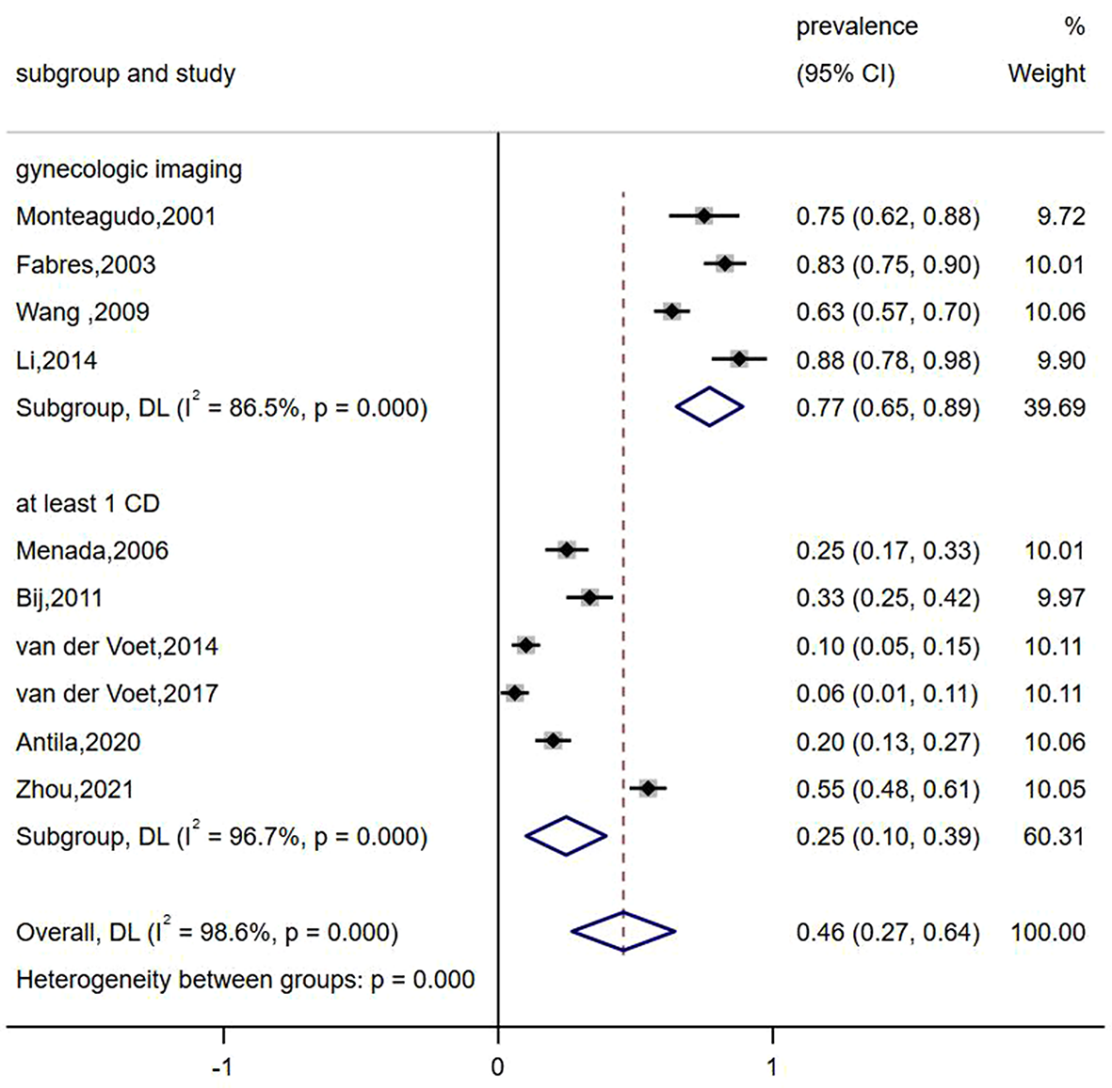
Subgroup analysis of the prevalence of abnormal uterine bleeding in patients with Cesarean scar defect

**Fig. 5:**
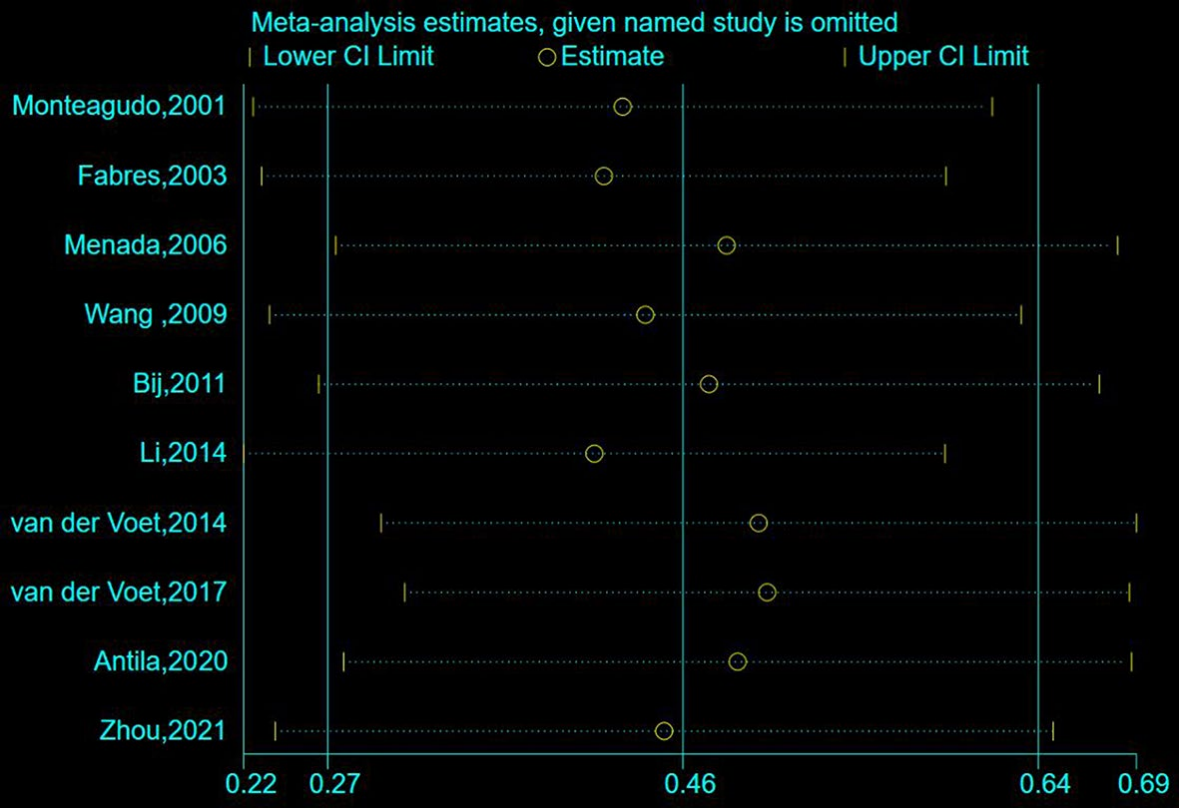
Sensitivity analysis of the prevalence of abnormal uterine bleeding in patients with Cesarean scar defect

Data on the prevalence of abnormal uterine bleeding in women with caesarean scar defects were reported in 10 studies, and subgroup analyses were conducted on different subgroups of study populations (women with at least one caesarean section; women with previous caesarean section who underwent imaging specifically for gynaecological diseases) to determine the prevalence of abnormal uterine bleeding in different study populations. The heterogeneity evaluation results indicated heterogeneity among the included articles (*I*^*2*^ = 98.6%, *P* < 0.001), and a meta-analysis was performed using a random-effects model, which revealed that the prevalence of abnormal uterine bleeding was around 0.46 (95% CI: 0.27–0.64). In the different populations, the prevalence of abnormal uterine bleeding in women undergoing imaging (0.77, 95% CI: 0.65–0.89) was significantly higher than that in women with at least one caesarean Sect. (0.25, 95% CI: 0.10–0.39), as shown in Fig. 4. The sensitivity analysis revealed a prevalence of 0.22–0.69 for abnormal uterine bleeding (see Fig. 5).

### Risk of intermenstrual bleeding

**Fig. 6:**
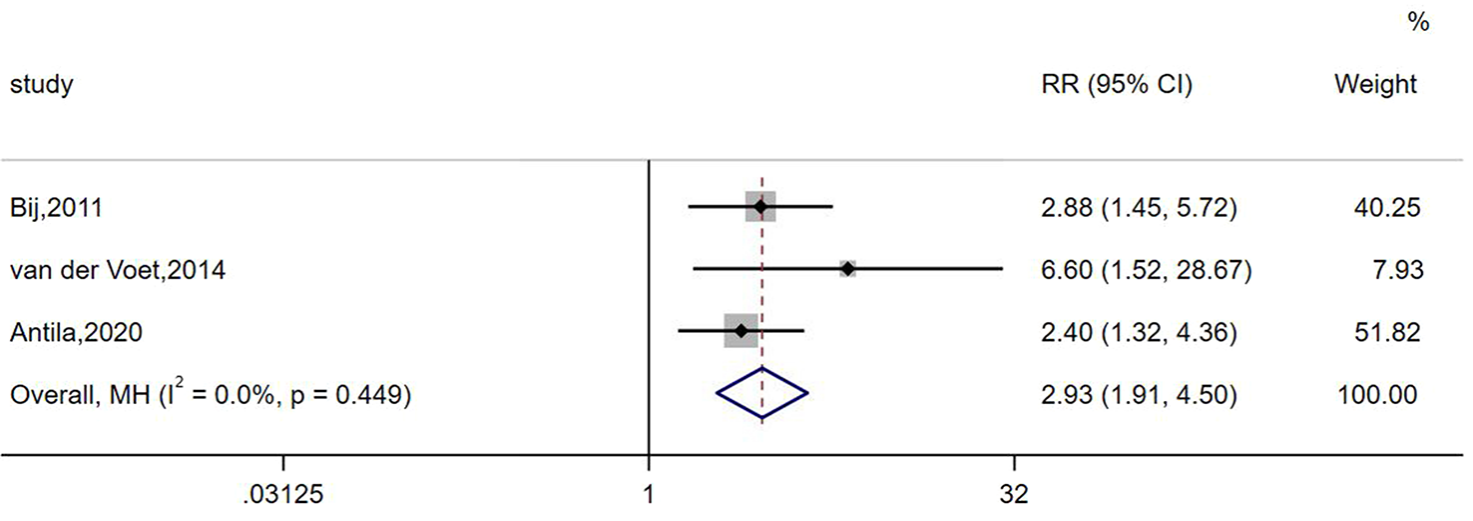
Forest plot of the relative risk of intermenstrual bleeding in patients with Cesarean scar defect versus those without

Three studies provided data on the risk of intermenstrual bleeding in women with caesarean scar defects. The heterogeneity results indicated no heterogeneity among the included articles, and the pooled effect size was calculated using a fixed-effects model. In addition, the meta-analysis indicated that women with caesarean scar defects were more likely to experience intermenstrual bleeding (RR: 2.93, 95% CI: 1.91–4.50) (see Fig. 6).

## Discussion

The association between caesarean scar defects and abnormal uterine bleeding was investigated in this study based on a systematic review and meta-analysis. The study designs of the 10 eligible articles were mainly cohort studies and cross-sectional studies, with a lack of data from prospective studies. However, the findings of our study still provide an evidence-based indication for clinical practice in this field, as well as evidence of causal inference between caesarean scar defects and abnormal uterine bleeding. The study findings suggest that women with caesarean scar defects face a significantly increased risk of abnormal uterine bleeding and intermenstrual bleeding, with caesarean scar defects being a risk factor for abnormal uterine bleeding.

Abnormal uterine bleeding is the most common symptom of caesarean scar defects, with the common bleeding pattern being persistent spotting after regular menstruation, and the total bleeding time per cycle usually lasts for over 10 days. Despite its relatively small volume, the diverticulum can still cause prolonged bleeding. Morris et al. [12] concluded that the inflammatory state of the endometrial tissue covering the diverticulum and local endometrial coagulation disorders are among the causes of abnormal uterine bleeding. The connective tissue at this site can also be replaced by the basal tissue, which may negatively affect contractile haemostasis. Moreover, data about histological case characteristics also provide solid evidence for this hypothesised cause of bleeding, and the most common histopathological features of Cesarina scar defects include fibrosis [26], necrotic tissue [27], endometriosis/adenomyosis [28] and inflammatory infiltration [29].

Regarding a dose-response relationship, there may be a strong relationship between caesarean scar defects and abnormal uterine bleeding. Previous studies have demonstrated that the risk of bleeding may be higher in large-sized defects. Specifically, large-sized caesarean scar defects with a depth of over 50% of the muscular layer (or a residual muscular layer thickness of below 2.2 mm) account for 11–45% of all scar defects, while the probability of abnormal uterine bleeding in defects with a depth of over 50% of the muscular layer is six times higher than that in the population with a depth of below 50% [22]. In addition, previous studies have shown that a residual muscular layer thickness of below 2.15 mm at the scar site is an independent risk factor for total menstrual bleeding time longer than 14 days [30] and that repair surgery for caesarean scar defects can improve bleeding symptoms [31, 32].

The cause of a caesarean scar niche appears to be multifactorial and likely a combination of technical factors (low incision location), anatomical factors (uterine retroflexion) and patient factors (body mass index, smoking, maternal age), which might impair healing [33]. In pregnancy, caesarean scar niches have been associated with placenta accreta spectrum disorder and uterine rupture [34]. It should be noted that the standard treatment of caesarean scar defects still requires further research. Caesarean scar defect is a critical problem, much like other uterine surgeries [35, 36], which can cause poor outcomes; therefore, exploring the relationship between caesarean scar defects and poor outcomes is necessary.

Our study provides updated research to explore the relationship between caesarean scar defects and abnormal uterine bleeding, and the quantitative analysis results provide indications for clinical practice [37]. However, it should be noted that this study comes with some limitations. Firstly, there were variations in the definitions of caesarean scar defects and abnormal uterine bleeding among the included articles, which may be one of the main factors contributing to the heterogeneity among them. Therefore, caution should be exercised when interpreting the findings of this study. Additionally, due to limited information and data from the original studies, the quantitative analysis was only conducted on the risk, prevalence and intermenstrual bleeding risk of abnormal uterine bleeding, without any quantitative analysis of other aspects of abnormal uterine bleeding.

In conclusion, the current study findings indicate a clear association between caesarean scar defects and abnormal uterine bleeding. In light of the limitation of this study regarding varied definitions of abnormal uterine bleeding, an expert consensus should be reached in the future to standardise key definitions in this field to ensure the credibility of the reported results. Furthermore, more high-quality prospective studies are still needed for further in-depth investigations into the association between caesarean scar defects and abnormal uterine bleeding.

## Data Availability

The datasets used and analyzed during the current study are available from the corresponding author on reasonable request.
